# Human Uterine Wall Tension Trajectories and the Onset of Parturition

**DOI:** 10.1371/journal.pone.0011037

**Published:** 2010-06-23

**Authors:** Peter Sokolowski, Francis Saison, Warwick Giles, Shaun McGrath, David Smith, Julia Smith, Roger Smith

**Affiliations:** 1 Mothers and Babies Research Centre, Hunter Medical Research Institute, University of Newcastle, John Hunter Hospital, Newcastle, Australia; 2 School of Electrical Engineering and Computer Science, University of Newcastle, Callaghan, Australia; 3 Faculty of Engineering, Computing and Mathematics, University of Western Australia, Crawley, Australia; Johns Hopkins University, United States of America

## Abstract

Uterine wall tension is thought to be an important determinant of the onset of labor in pregnant women. We characterize human uterine wall tension using ultrasound from the second trimester of pregnancy until parturition and compare preterm, term and twin pregnancies. A total of 320 pregnant women were followed from first antenatal visit to delivery during the period 2000–2004 at the John Hunter Hospital, NSW, Australia. The uterine wall thickness, length, anterior-posterior diameter and transverse diameter were determined by serial ultrasounds. Subjects were divided into three groups: women with singleton pregnancies and spontaneous labor onset, either preterm or term and women with twin pregnancies. Intrauterine pressure results from the literature were combined with our data to form trajectories for uterine wall thickness, volume and tension for each woman using the prolate ellipsoid method and the groups were compared at 20, 25 and 30 weeks gestation. Uterine wall tension followed an exponential curve, with results increasing throughout pregnancy with the site of maximum tension on the anterior wall. For those delivering preterm, uterine wall thickness was increased 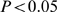 compared with term. For twin pregnancies intrauterine volume was increased compared to singletons (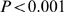), but wall thickness was not. There was no evidence for increased tension in those delivering preterm or those with twin gestations. These data are not consistent with a role for high uterine wall tension as a causal factor in preterm spontaneous labor in singleton or twin gestations. It seems likely that hormonal differences in multiple gestations are responsible for increased rates of preterm birth in this group rather than increased tension.

## Introduction

During pregnancy the uterus grows from a 70-gram near solid organ with a narrow cavity to a hollow tubular organ weighing 1100 grams. It accommodates an average total of 5 liters at term compared to 10 milliliters in the non-pregnant state [1]. The changes in the uterus during the first trimester are mainly due to the effects of estrogen and progesterone, not the effects of uterine distension created by the growing fetus [2] as the same uterine changes were found in pregnancies implanted outside the uterus.

An effect of uterine stretch on the duration of human pregnancy has been suggested by the strong association of multiple gestations with preterm delivery [3]–[6]. Gardner et al. [5] found that 54% of twin pregnancies were delivered preterm compared to only 9.6% who were delivered preterm in a comparison singleton pregnancy group. Minakami et al. [6] studied the risk of premature birth in multifetal pregnancy by analyzing 6,036,475 singleton pregnancies and 90,887 multifetal pregnancies in Japan and concluded that the risk of preterm delivery is nine times higher in women with multifetal pregnancies than in singleton pregnancies. Although the mechanism was uncertain, it appeared possible that uterine stretch played a key role in the increase of preterm birth in multifetal pregnancy.

Uterine stretch is measured as the amount of tension exerted on the wall of the uterus. It is used interchangeably with myometrial tension and uterine wall tension. Myometrial tension is the force per unit area that resists pressure across the uterus. The volume of the uterus, the intrauterine pressure, the thickness of the uterine wall and its surrounding support determine myometrial tension [7], each variable being individually analyzed in the past but seldom used in combination to determine myometrial tension thus little work has been conducted on myometrial tension in human pregnancy. A study by Ulmsten et al. [8] dealt with intrapartum myometrial tension in term, non-laboring women where induction of labor was achieved with oxytocin infusion. They reported that women with low myometrial tension failed induction of labor. Anderson et al. [7] investigated the postpartum uterus removed for postpartum haemorrhage. Myometrial tension was calculated using a graded pressure and they showed that the myometrial tension was directly proportional to amniotic fluid pressure. In the studies cited above, myometrial tension was calculated based on the Law of Laplace [9] and assumed a spherical uterus.

At present, there is no reported study on myometrial tension throughout a normal pregnancy in humans. We sought to provide this data. As data on intrauterine pressure in both singletons and twins was already available in the literature we did not consider it ethical to repeat these measurements; we therefore used pressure measurements throughout gestation from the literature combined with our ultrasonography measures of uterine size, shape and wall thickness throughout pregnancy to construct a uterine wall tension trajectory based on a prolate ellipsoid as a better approximation of the uterine shape [10]. Our specific objectives were to (a) characterize uterine wall thickness, intrauterine volume and maximum uterine wall tension trajectories from 20 weeks gestation to delivery for preterm and term singletons with spontaneous labor onset and for twin pregnancies and (b) assess group differences (preterm versus term, twin versus term) in uterine wall thickness, intrauterine volume and maximum uterine wall tension at 20, 25 and 30 weeks gestation.

## Materials and Methods

### Ethics Statement

This study was conducted according to the principles expressed in the Declaration of Helsinki. Both the Human Ethics committee of the Hunter Area Health Service and the Human Research Ethics Committee of the University of Newcastle approved this study and all subjects provided written informed consent for the collection of samples and subsequent analysis.

### Study Design

**Figure 1:**
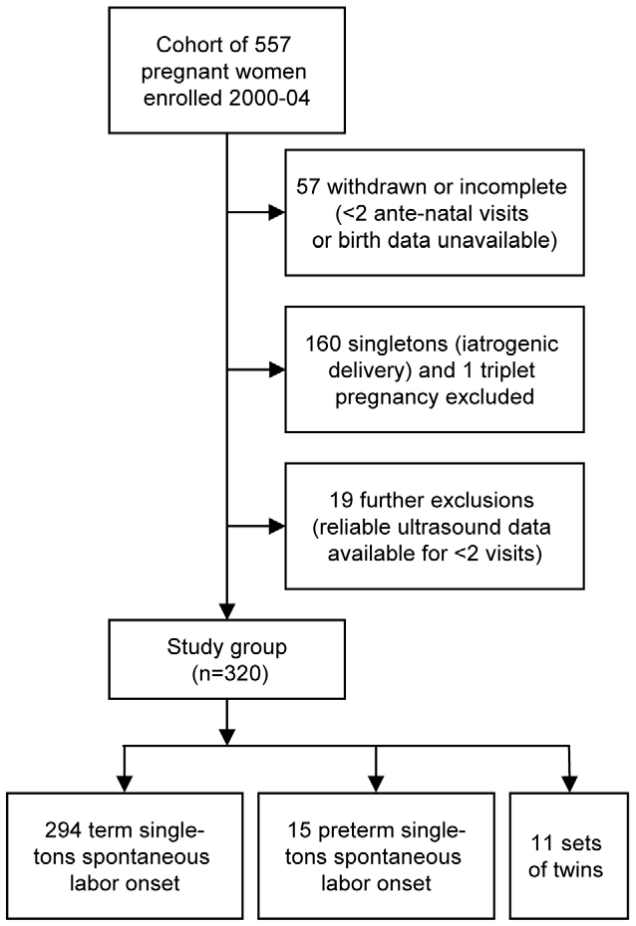
Flowchart of study enrolment and exclusions.

This was a longitudinal study where unselected subjects were recruited by research midwives at their first antenatal visit and followed to delivery at the John Hunter Hospital in Newcastle, Australia, during the period 2000–2004. Ultrasound readings were taken at up to four antenatal visits. A cohort of 557 women were recruited of which term singleton and preterm singleton deliveries that underwent induction of labor or Cesarean section were excluded, and after withdrawals formed a study group of 320 women (Figure 1). Gestational length at delivery and gestational age at the time of ultrasounds were based on early ultrasound scans. The women were predominately Caucasian (92%), with a small percentage of Aboriginal or Torres Strait Islander descent (3%) and others including Asian descent (5%). These women were followed until delivery and routine clinical data were collected including maternal smoking status, birth weight and sex of the fetus. The preterm delivery rate was 7.2% in singleton gestations and 72% in twin gestations (preterm delivery was defined as gestational length less than 37 weeks).

### Instrumentation

The schedules of ultrasound assessments were at 20, 24, 30 and 36 weeks gestation during scheduled prenatal visits, although the actual timing of assessments may have varied due to schedule availability as shown in Table 1. The first ultrasound assessment at 20 weeks included a fetal morphology study and confirmation of gestational age. Ultrasonographic measurements were performed with an Aspen Advanced Ultrasound System (Acuson, Mountain View, CA) using a 1.75–4.0 MHz transducer. The machine has Freestyle™ extended imaging capability that provides expansive anatomical views and allows the scanner to see panoramic images of the uterus quickly with zoom, rotate, and measurement capabilities. The built-in computer system analyzes the images in the frame, composes and displays them as an extended field-of-view image. A Freestyle™ extended image is constructed from the sequence of image frames captured from the transducer as it moves along the long axis of the uterus. The computer uses mathematical algorithms to convert the image frames into a single extended image. Note that the entire uterus can be visualized from the fundus to the cervix, the anterior to posterior wall are well delineated and can be measured using electronic callipers.

**Table 1:** Maternal, Fetal and Pregnancy Characteristics.

Study Group (320 women)		Twin pregnancies ( 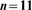 )	Preterm singleton pregnancies ( 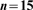 )	Term singleton pregnancies ( 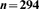 )
Maternal age (yrs)†		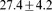	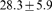	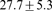
Primiparous		3 (27%)	3 (20%)	145 (49%)
No. of ultrasound scans†		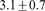	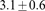	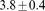
Scan week†:	1st scan	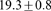	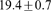	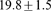
	2nd scan	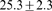	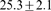	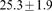
	3rd scan	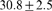	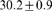	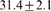
	4th scan	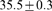	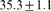	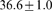
Maternal morbidities:	E. hypertension‡	0	0	0
	G. hypertension‡	4	0	0
	Pre-eclampsia	1	0	0
	Gestational diabetes	0	0	0
Smoking (self-reported):	at enrolment	2 (18%)	10 (67%)	71 (24%)
	at delivery	2 (18%)	8 (53%)	66 (22%)
Gestation at delivery†		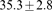	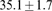	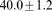
Fetal sex:	Females	11 (50%)	10 (67%)	153 (52%)
	Males	11 (50%)	5 (33%)	141 (48%)
SGA*		4 (18%)	0 (0.0%)	28 (9.5%)
Birthweight (g)†		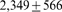	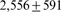	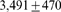

† Mean 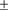 SD.

‡ denotes Essential hypertension and Gestational hypertension respectively.

* Small Gestational Age (SGA) if under the 10th centile for gestational age [23].

**Figure 2:**
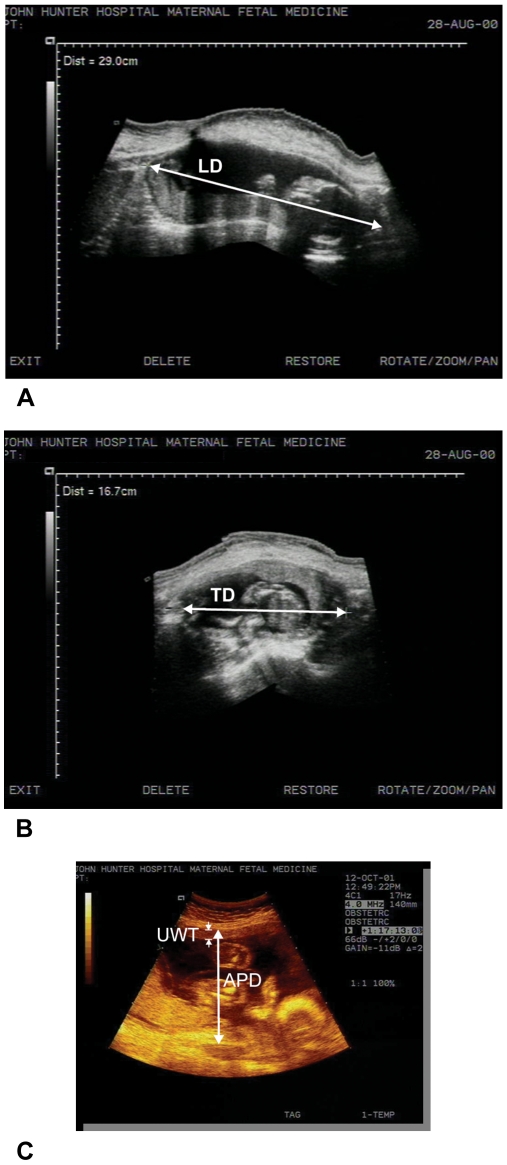
Examples of ultrasonographic measurements taken with an Aspen Advanced Ultrasound System. (**A**) Longitudinal Diameter (LD) of the uterus using Freestyle™ software; (**B**) Transverse Diameter (TD) of the uterus using Freestyle™ software; (**C**) Anterior-Posterior Diameter (APD) and Uterine Wall Thickness (UWT) shown.

The transducer was taken longitudinally from the uterine fundus and swept downward to the cervix. Using the image of the whole uterus the length of the uterus, also referred to as the longitudinal diameter (LD), was measured from the outer margin of the fundus to the cervix (Figure 2A). With the same image, the widest anterior-posterior diameter (APD) of the uterus was measured from the outer margins of the anterior surface to the posterior surface perpendicular to the longitudinal diameter. The anterior-posterior diameter is shown in the image of Figure 2C. For the transverse diameter (TD), the transducer was rotated ninety degrees and then placed from one side of the uterus at its maximal width until the outer margin could be visualized. The transducer was then swept to the other side making sure the outer margin could also be seen. The widest transverse diameter from the outer margins was measured as the transverse of the uterus, shown in Figure 2B.

### Measurement of Uterine Wall Thickness and Calculation of Intrauterine Volume

Ultrasonographic evaluation of the uterine wall was carried out with an empty bladder. The thickness of the anterior uterine wall (UWT) was measured at the level of maximum anterior-posterior diameter, that is at the most anterior position (Figure 2C). The wall was defined as a layer of echogenic signal from the serosa to the decidua.

Calculation of total intrauterine volume (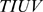) was based on the assumption that the shape of the uterus is a prolate ellipsoid. It was measured using the Prolate Ellipsoid (PE) method where volume is equal to the product of the length (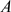), the transverse diameter (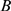), the anterior-posterior diameter (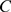) of the uterus and a constant (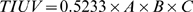). Some authors [11], [12] attributed the inaccuracies of the PE method to distension of the urinary bladder and poor visualization of the uterine outline. In this study the bladder was emptied and the Freestyle™ extended imaging capability of the ultrasound machine provided clear and distinct visualization of the uterine margins.

### Calculation of Uterine Wall Tension

**Figure 3:**
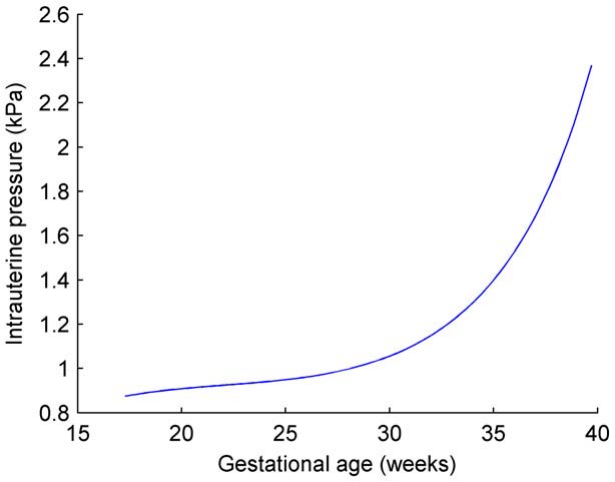
Intrauterine pressure at gestational age 17 to 40 weeks. IUP was interpolated and extrapolated from Fisk et al. [15].

The determinants of uterine wall tension are: (a) uterine wall thickness, (b) intrauterine volume and (c) intrauterine pressure. The accepted method for measuring intrauterine pressure (IUP) is the direct method that requires insertion of a catheter or a transducer into the intrauterine cavity via the transvaginal or transabdominal approach [13]. The availability of this data in the literature and the complications that may occur with any procedure entering the intrauterine cavity prevented its use in this study. Such complications include: injury to the fetus, preterm labor, rupture of membranes, intrauterine infection, placental abruption, and uterine perforation. This also explains the limited literature regarding the measurement of IUP throughout pregnancy. A new non-invasive method for measuring IUP has been established by Skowronski et al. [14] in 2006 but is not yet in common usage and was unavailable at the time of the study. An investigation by Fisk et al. [15] reported the use of direct measurement of IUP throughout pregnancy. They showed a significant increase of IUP with gestation (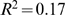 and 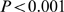). Values for mean IUP were both extrapolated and interpolated from the study by Fisk et al. [15]. These values are shown in Figure 3 and were generated by using the best fitted regression line which is a fifth order polynomial regression and was performed after conversion from mm of Hg to Pascals, the metric system of measurement for pressure. Fisk et al. also provided data on IUP in multiple gestations which were not significantly different from those in singletons.

The Law of Laplace relates pressure difference to the curvature of a surface, and its surface tension [16]. It may be written as:
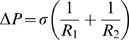
(1)where 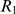 and 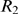 are the radii of curvature of the surface along any two orthogonal tangents (principal radii of curvature), and 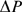 is the difference in fluid pressure across the curved surface. The quantity 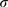 is called the **surface tension** and is usually given in units of force per unit length. The surface tension may further be expressed as the product of the wall thickness 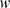, and wall tension per unit cross section of wall 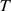, that is, 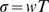.
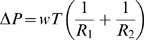
(2)For a surface, the curvature is the absolute value of the rate of change of the angle of inclination of the tangent line with respect to distance along the curve. In rectangular Cartesian coordinates, the curvature 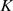 is given by
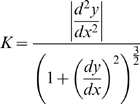
(3)The curvature 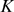 is inversely related to the radius of curvature such that 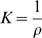 [17]. Thus, pressure difference may now be written as:
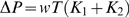
(4)where 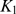 and 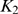 are the **curvatures of the surface**.

Assuming the shape of the uterus is closely related to that of an ellipsoid then the equation to describe the surface of the uterus in rectangular Cartesian coordinates is
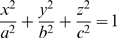
(5)where 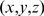 is any point on the surface of the uterus, and 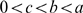 with 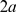, 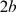 and 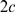 being the lengths of the 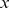-axis, 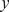-axis and 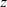-axis respectively. It would be usual to implicitly differentiate equations 3 and 5 to find the two 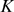 values 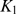 and 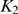 but in this case we need to find the curvature of the surface, not the radial curvature. Here we need to make use of Differential Geometry to find these values of 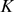.

Note that an ellipsoid is a level surface [18]. Let 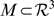 be a smooth surface given as 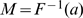 for some smooth function 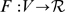, where 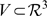 is an open set and 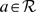 is a number. Fix a point 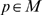. It can be shown that the **mean curvature** 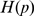 at 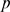 of the function 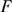 is:
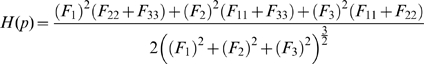
(6)assuming that all the mixed second partial derivatives of 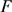 are constantly zero, that is 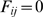 for 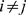. Note, the notation 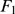 means the first partial derivative with respect to the first coordinate, 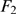 the first partial derivative with respect to the second coordinate, and 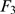 the first partial derivative with respect to the third coordinate. Similarly, 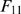 means the second partial derivative with respect to the first coordinate, and so on. But the definition of mean curvature is
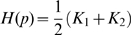
(7)where 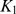 and 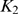 are the same 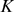
s as in equation 4. So using equations 6 and 7 and letting the function 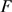 be that defined in equation 5 we can find the sum of 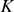 to substitute into equation 4 and thus find an expression for the tension of the myometrium. That is, let
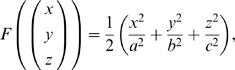
then
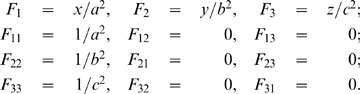
These are all the partial derivatives needed to compute 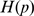 and we do note that 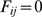 for 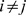, so this fits our original assumption. Now substituting these into equation 6 we have
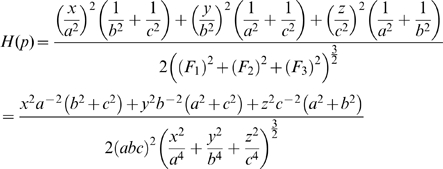
Now from equation 7 we can find the sum of the curvatures of the surface as a function of the coordinates 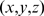, that is:
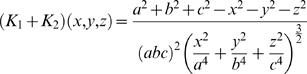
It can be shown that this sum is a minimum when 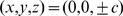, that is where the ellipsoid surface is the flattest. Hence, from equation 4, the tension is at its maximum at this point. So
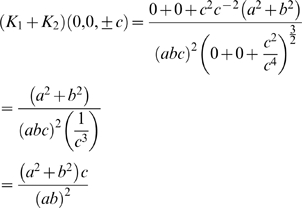
Thus, substituting this into equation 4 and rearranging, the maximum tension of the myometrium is
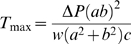
(8)where 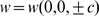, that is, 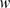 is the uterine wall thickness at this flattest point. We assume the anterior lower segment wall thickness to be this value because the wall thickness is relatively uniform and proportionality will hold.

### Modeling Trajectories of Growth and Myometrial Tension

MATLAB® Statistics Toolbox Version 4.0, Release 13 (The Mathworks Inc., Natick, MA) was used for curve-fitting and statistical analysis. Results for the variables uterine wall thickness, intrauterine volume and maximum myometrial tension were curve-fitted to provide trajectories through to parturition for each individual pregnancy. These trajectories were used to provide group comparisons as well as mean and percentile curves; this method also partially reduces the effect of measurement error in individual ultrasound measurements. A single equation type was assumed to curve-fit the samples for each variable and lower order polynomial or exponential equations were preferred. A few plausible candidate equations were chosen for each variable (guided by scatter plots and line graphs of each individual for all groups) and fitted to the data for each subject by non-linear least squares estimation where goodness of fit was evaluated. A final choice of equation was made for each variable, primarily by consideration of normality and homoscedasticity in the residuals. The equations and individual stored coefficients 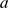 and 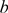 for each subject and each variable enabled wall thickness, intrauterine volume and maximum uterine wall tension to be calculated.

### Statistical Analysis

Hypothesis tests of group medians were conducted using Wilcoxon rank-sum non-parametric statistical tests, as appropriate to the distribution of the data. The term singleton group was compared with the preterm singleton group and the twin group at 20, 25 and 30 weeks gestation. These time steps were chosen to ensure all trajectories encompass the analyses, and to ensure no preterm trajectories were omitted. A two-tailed significance level of 5% was used throughout.

## Results

A total of 294 spontaneous term singleton, 15 spontaneous preterm singleton and 11 twin pregnancies which satisfied our criteria were studied. The earliest ultrasound measurement was at 17 weeks and the latest at 39 weeks. Total gestation ranged from 37–44 weeks for term singletons, 31–36 weeks for preterm singletons and 29–38 weeks for twins. Further maternal, fetal and pregnancy characteristics are provided in Table 1.

Uterine wall thickness was approximately constant across gestation for each pregnancy with a very slight decline in later weeks in some trajectories. Intrauterine volume and uterine wall tension were monotonic increasing. The following equations were chosen:
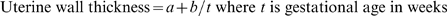
(Mean values of the Coefficient of Determination (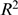) for goodness of fit to uterine wall thickness in the three groups were: Twin 67%, Preterm 71%, Term 61%)
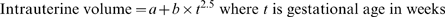
(Mean 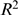 for intrauterine volume: Twin 99%, Preterm 99%, Term 98%)
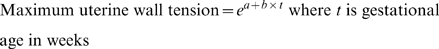
(Mean 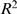 for maximum uterine wall tension: Twin 79%, Preterm 80%, Term 88%)

**Figure 4:**
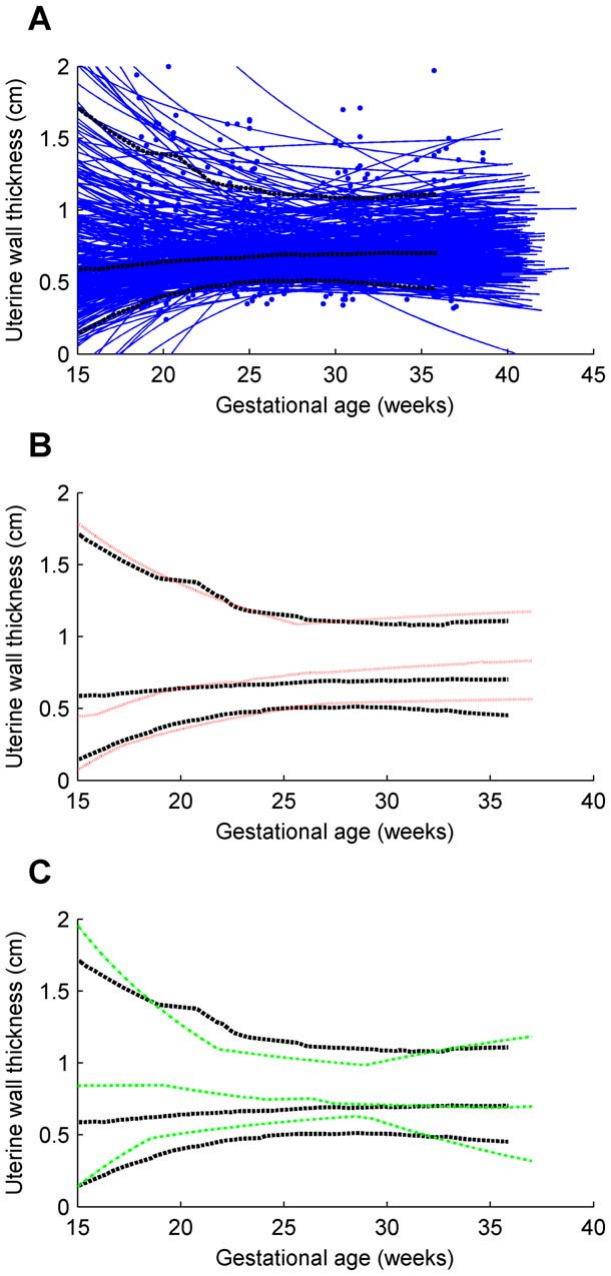
Uterine wall thickness trajectories for term singleton, preterm singleton and twins. (**A**) term singleton trajectories and their 5th, 50th and 95th centiles; (**B**) preterm singletons 5th, 50th and 95th centiles shown against corresponding term singletons centiles; (**C**) twins 5th, 50th and 95th centiles shown against corresponding term singletons centiles. *Series of blue dots fitted with blue solid lines*, term singleton trajectories; *black bold dashed lines* 5th, 50th and 95th centiles of term singletons; *red dotted lines* 5th, 50th and 95th centiles of preterm singletons; *green dot-dashed lines* 5th, 50th and 95th centiles of twins.

**Figure 5:**
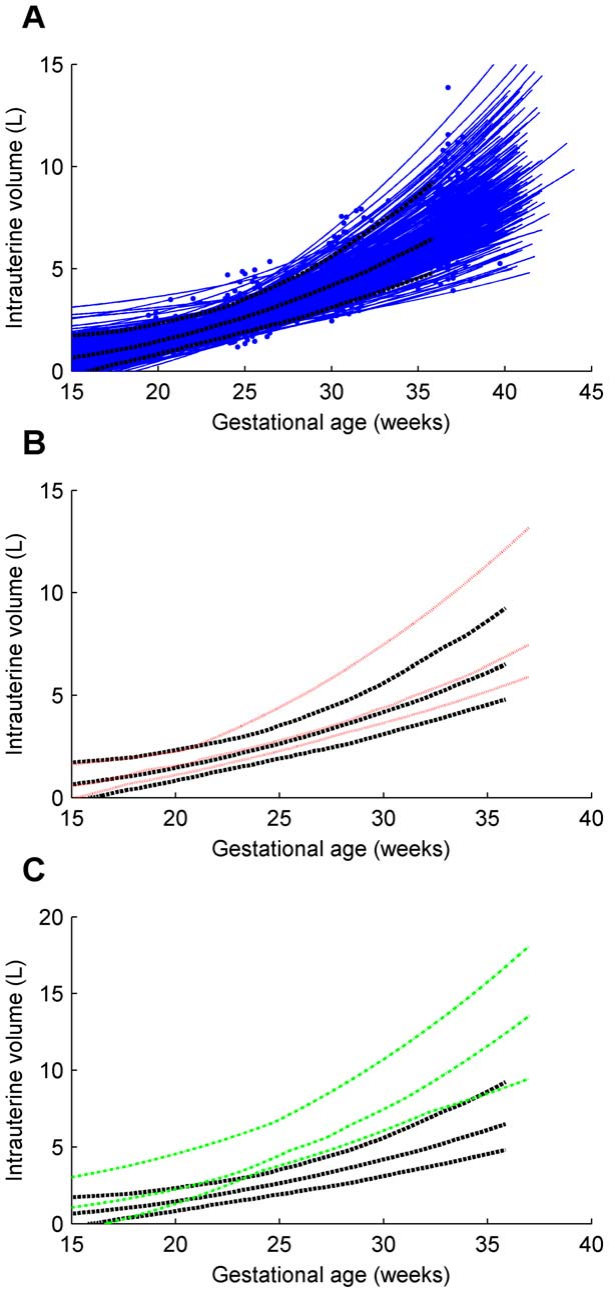
Intrauterine volume for term singleton, preterm singleton and twins. (**A**) term singleton trajectories and their 5th, 50th and 95th centiles; (**B**) preterm singletons 5th, 50th and 95th centiles shown against corresponding term singletons centiles; (**C**) twins 5th, 50th and 95th centiles shown against corresponding term singletons centiles. *Series of blue dots fitted with blue solid lines*, term singleton trajectories; *black bold dashed lines* 5th, 50th and 95th centiles of term singletons; *red dotted lines* 5th, 50th and 95th centiles of preterm singletons; *green dot-dashed lines* 5th, 50th and 95th centiles of twins.

**Figure 6:**
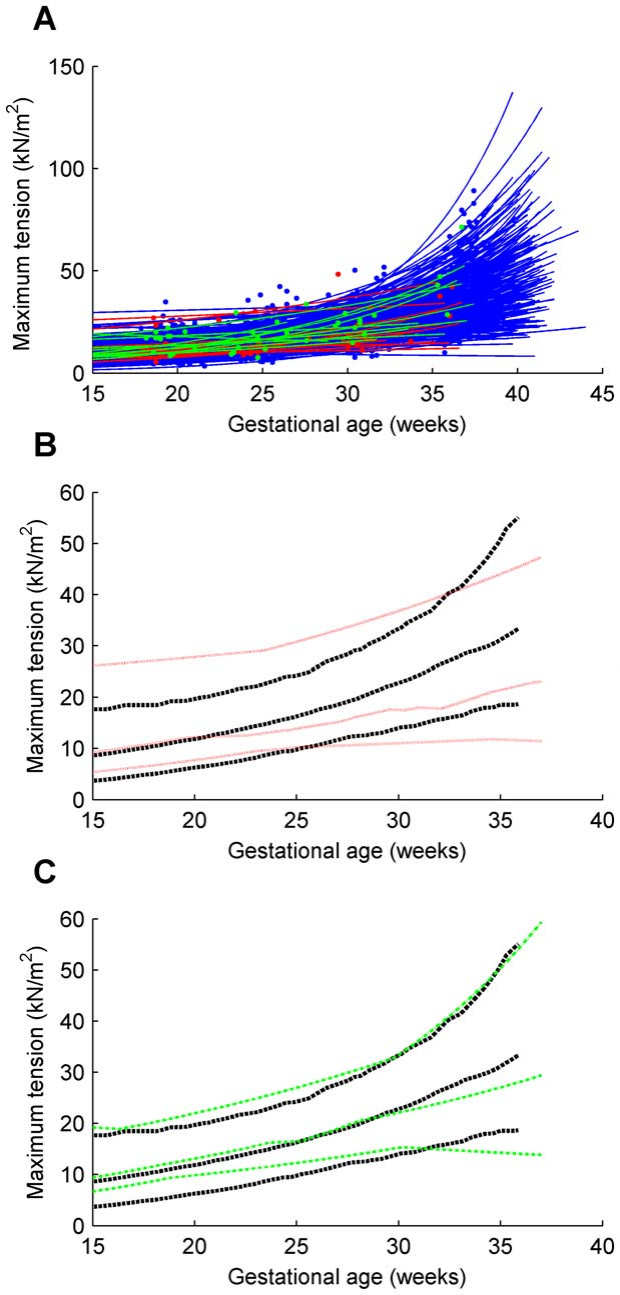
Maximum uterine wall tension for term singleton, preterm singleton and twins. (**A**) term singleton, preterm singleton and twin trajectories; (**B**) preterm singletons 5th, 50th and 95th centiles shown against corresponding term singletons centiles; (**C**) twins 5th, 50th and 95th centiles shown against corresponding term singletons centiles. *Series of blue dots fitted with blue solid lines*, term singleton trajectories; *series of red dots fitted with red solid lines*, preterm singleton trajectories; *series of green dots fitted with green solid lines*, twin trajectories; *black bold dashed lines* 5th, 50th and 95th centiles of term singletons; *red dotted lines* 5th, 50th and 95th centiles of preterm singletons; *green dot-dashed lines* 5th, 50th and 95th centiles of twins.

Individual trajectories and the 5th, 50th and 95th centile curves for uterine wall thickness are shown for the twin, preterm and term groups in Figure 4. Similarly, intrauterine volume and maximum uterine wall tension are shown in Figures 5 and 6 respectively. The Materials and Methods section shows the maximum tension of the uterine wall occurred at 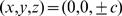. That is, on either the anterior or posterior wall of the uterus. The prolate ellipsoid model used for the uterine shape achieves the correct curvature at the anterior. However, the model does not mimic the curvature at the posterior due to the uterus being pushed against the spine but since the curvature at the posterior is smaller than the anterior this means the maximum tension of uterine wall only occurs at the anterior, that is when 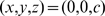.

**Figure 7:**
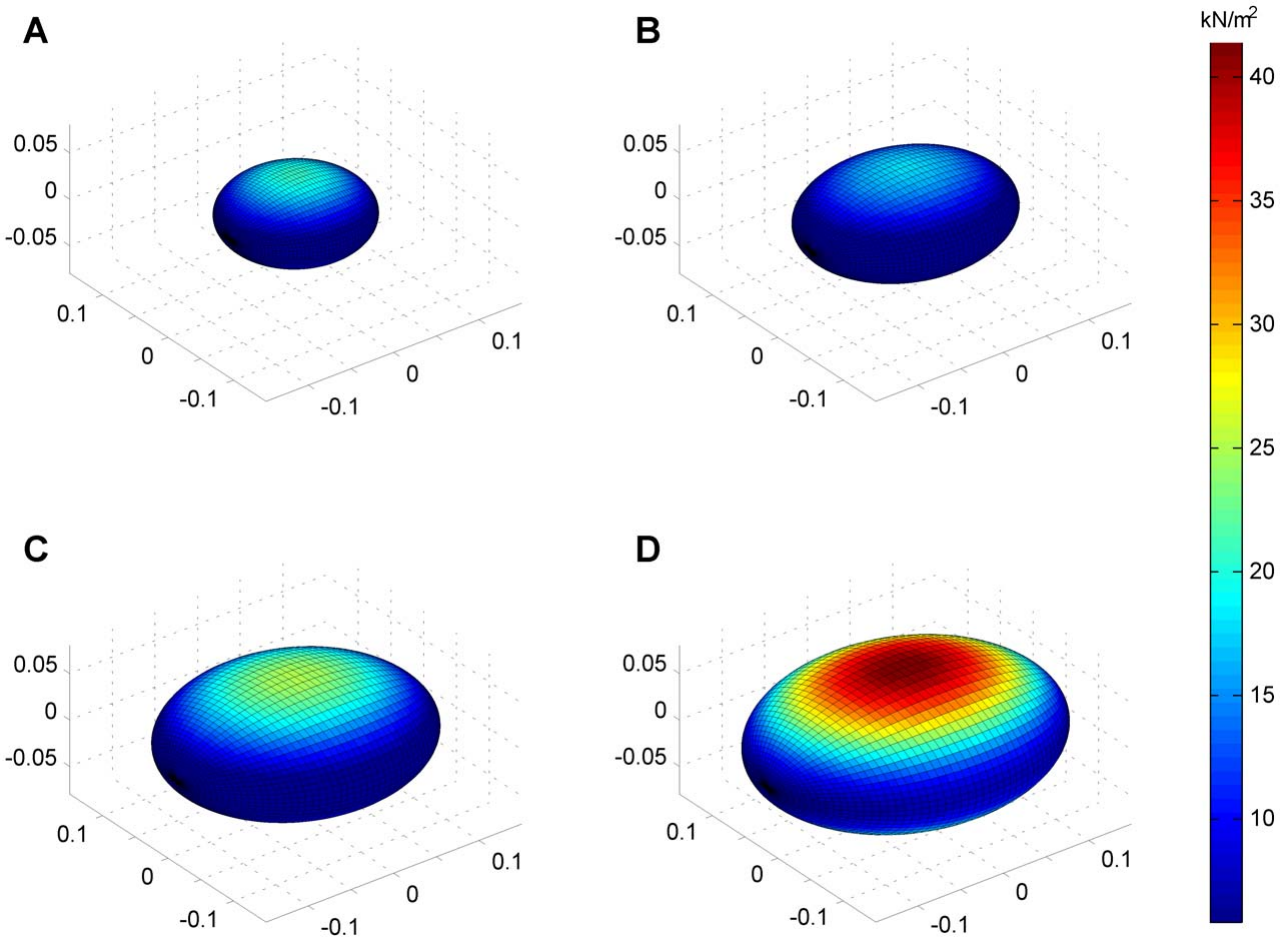
The developed equations for human uterine wall tension show a developing human uterus growing throughout gestation. Maximal tension occurs at the anterior and posterior of the uterus. The 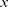, 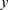, 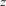 axes are in meters, and the color bar describes uterine wall tension. (**A**) gestational age of 19 weeks; (**B**) gestational age of 25 weeks; (**C**) gestational age of 30 weeks; (**D**) gestational age of 36 weeks.

The tension derivation can be easily understood by visually examining diagrams of the developing human uterus growing throughout gestation and how it varies for each point on the wall of the uterus, shown in Figure 7. Note as gestational age increases, the change in color indicates an increase in uterine tension, and the maximum tension (at the anterior) increases exponentially.

Uterine wall thickness, intrauterine volume and maximum uterine wall tension were interpolated from the trajectory equations for each subject at 20, 25 and 30 weeks and non-parametric tests for group medians were conducted. The results (in Table 2) show that median uterine wall thickness for the preterm group is significantly higher than that for the term group at 30 weeks. Intrauterine volume is significantly higher in twins than in term singletons at all three time points. Maximum uterine wall tension in twins is no different from that in term singletons but maximum uterine wall tension in preterm singletons is significantly lower than that in term singletons by 30 weeks.

**Table 2:** Results of hypothesis tests comparing twin, preterm and term groups.

Variable	Gestation week	Twin median 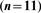	Preterm median 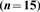	Term median 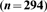	Twin vs Term 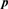 value	Preterm vs Term 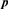 value
Uterine wall thickness (cm)	20	0.82	0.64	0.64	0.08	0.73
	25	0.75	0.73	0.67	0.07	0.31
	30	0.71	0.78	0.69	0.47	0.05
Intrauterine volume (L)	20	2.232	1.558	1.459	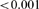	0.40
	25	4.452	2.789	2.637	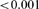	0.12
	30	7.459	4.406	4.189	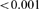	0.09
Maximum uterine wall tension (kN 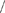 m 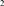 )	20	13.1	12.2	11.9	0.11	0.47
	25	16.5	13.9	16.3	0.33	0.23
	30	22.1	17.5	22.8	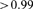	0.002

## Discussion

The results for uterine wall thickness are similar to those of Degani et al. [19] who also found an initial increase during mid-pregnancy in the thickness of the anterior uterine wall where these measurements were taken. However, in their study after the second trimester there was a significant decline in the thickness of the myometrium in the anterior aspect of the lower uterine segment, which was not examined in our study. In our study, wall thickness did not change significantly across pregnancy in singleton or twin pregnancies while the preterm group showed an increase. Interestingly Buhimschi et al. [20] have also reported an increase in myometrial thickness in subjects with premature rupture of membranes. In their analysis increased myometrial thickness was associated with a longer latency to delivery. No such relationship was evident in our data. In premature rupture of membranes a loss of amniotic fluid may be expected to result in a reduced intrauterine volume; to emphasize the difference in our own setting of spontaneous preterm birth no reduction in intrauterine volume was observed.

A greater range of uterine wall thickness was observed in the earlier stages of pregnancy which may relate to increased measurement error at this gestational age; from 26 weeks gestation until delivery results were remarkably stable. These data suggest that myometrial growth effectively compensates for the increasing intrauterine contents, maintaining a constant uterine wall thickness. Sfakianaki et al. [21] observed a reduction in uterine wall thickness in twin gestations at the lower uterine segment just above the bladder. We did not specifically assess this site but examined the wall thickness at the site of likely maximum tension on the anterior surface of the uterus at the point of greatest posterior to anterior diameter.

For the total intrauterine volume, Gohari et al. [22] measured the TIUV using the PE method and their results showed a lesser volume compared to the outcome of this study. This difference may be because the population studied were at high risk for being born small for gestational age (SGA) [23] and 28% of the babies born in that population were classified SGA, whereas in our study population approximately 10% of the term singleton babies were SGA (as expected) but none of the preterm singletons was SGA. The TIUV shown in Figure 5 displayed a steady increase throughout pregnancy. The greater increase of TIUV in twin gestations relative to the term singletons group was evident from early in pregnancy and was highly significant. Our preterm group showed slightly greater volumes than the term singletons.

The graphs in Figure 6 show the maximum uterine wall tension throughout pregnancy. This followed an exponential function increasing until parturition. Results for twin gestations were not significantly different from that observed in singleton term deliveries. This surprising result suggests that factors other than increased tension are responsible for the increased rates of preterm birth in twin pregnancies. Twin pregnancies are known to have an altered endocrinology compared to singletons. Notably in twin gestations maternal plasma corticotropin-releasing hormone (CRH), progesterone, estradiol and estriol are all elevated [24]. The higher levels of CRH may drive preterm delivery in twin gestations. The data also suggest that increased tension is not a critical determinant of preterm birth in singleton gestations. In fact later preterm birth pregnancies were associated with a lower uterine wall tension that became statistically significant by 30 weeks. Our data is restricted to the late preterm group delivering after 32 weeks. Earlier preterm births are thought more likely to be due to infective or inflammatory etiologies [25] while late preterm births have been suggested to be due to disturbances in the gestational length mechanisms such as increased CRH production from the placenta. Nevertheless our data may indicate some inflammation leading to thickening of the uterine wall in cases of late preterm delivery, a consequence of which is reduced uterine wall tension.

Lye et al. [26] have shown that in rats, uterine stretch plays a key role in orchestrating the uterine changes that occur as the uterus becomes active. Myometrial activation from uterine stretch causing an increased excitability and responsiveness were shown to be due to increased expression of contraction associated proteins (CAPs) such as connexins, and prostaglandin receptors. The ability of uterine stretch to initiate uterine contractions was shown by Manabe et al. [27] who compared the changes in the levels of amniotic fluid and plasma prostaglandin F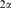 metabolites before and during uterine distension in pregnant women having a live or dead fetus. Prostaglandin F values were significantly correlated with the progress of labor (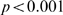) but there was no significant difference between live and dead fetuses.

Further evidence that stretch is important for uterine contraction was provided by a study from Wathes and Porter [28] on rat myometrium. They inserted an intrauterine balloon into ovariectomized post-partum rats. Estradiol 17B was given to experimental rats; the intrauterine pressures and gap junction formation were compared between experimental and control animals. Their results showed a significant increase in the number of gap junctions per mm of tissue and an increase in the rate of rise of pressure in the estrogen treated rats. There was also a significant increase in gap junctions formed in the balloon-filled uterine horn in the control rats. This implies that uterine distension without an estrogenic effect increases gap junction formation that is important for coordinated uterine contraction. Our data do not refute the potential role of increasing uterine stretch in initiating uterine contractions or increasing the force of such contractions. However the data show that when compared with the normal spontaneous term singleton delivery trajectories, there was no increase in maximum uterine wall tension trajectories in subjects pregnant with twins and moreover, in those with singleton pregnancies delivering preterm there was evidence for reduced tension from 30 weeks. This should direct research in these pathological groups away from increased tension as a critical determinant of gestational age at delivery.

Our data provide the first trajectories for maximum uterine wall tension across gestation in term singleton, preterm singleton and twin gestations. Our results are dependent on a number of assumptions which include data from the literature on intrauterine pressure in singletons and twin gestations that indicates similar pressures for both groups, and minimal effect of surrounding uterine wall support on our model. These assumptions may cause the magnitude of the reported uterine wall tension to vary slightly from the actual magnitude, however the shape and characteristic of the trajectories would remain much the same. The analysis shows a gradual rise in uterine wall tension in each of these groups. Myometrial tension in twin gestations was not increased relative to term singleton gestations. Further, a relative decrease in tension was observed in the preterm singleton gestations compared with the term singleton group. Therefore our data do not demonstrate a relative increase in uterine wall tension that might be a pathophysiological mechanism leading to preterm birth in twins or spontaneous preterm birth. The data surprisingly show a small but significant increase in uterine wall thickness in women delivering preterm with a singleton gestation. Modeling suggests that maximum uterine wall tension would occur at the anterior surface of the uterus as this is the site of maximum curvature (note the posterior surface is distorted by the vertebrae and therefore will have a lesser curvature and lower tension). If uterine wall tension plays a critical role in the initiation of uterine contractions these might be expected to be initiated in the areas under greatest tension anteriorly.
